# Adaptive noise canceling for transient absorption microscopy

**DOI:** 10.1117/1.JBO.25.10.106503

**Published:** 2020-10-14

**Authors:** Erkang Wang, Saurabh Gupta, Jesse W. Wilson

**Affiliations:** a Colorado State University, Department of Electrical and Computer Engineering, Fort Collins, Colorado, United States; b Colorado State University, School of Biomedical Engineering, Fort Collins, Colorado, United States

**Keywords:** pump-probe microscopy, transient absorption microscopy, signal processing, laser noise, relative intensity noise, balanced detection

## Abstract

**Significance:** Ultrafast fiber lasers are an attractive alternative to bulk lasers for nonlinear optical microscopy for their compactness and low cost. The high relative intensity noise (RIN) of these lasers poses a challenge for pump-probe measurements such as transient absorption and stimulated Raman scattering, along with modalities that provide label-free contrast from the vibrational and electronic structure of molecules.

**Aim**: Digital adaptive filtering was applied to determine the applicability for canceling laser RIN in a transient absorption microscope with an ultrafast fiber laser source.

**Approach**: Digitized signals from the transmitted probe and reference photodetectors were fed to an adaptive filter in MATLAB, running in a noise canceling configuration. This result was then fed to a software lock-in algorithm to demodulate the pump-probe signal. Images were built up one line scan at a time with a 3.5-kHz resonant scanner, with 100× averaging. The imaging target was ${\mathrm{Bi}}_{4}{\mathrm{Ge}}_{3}O_{12}$, which exhibits nondegenerate two-photon absorption at the pump/probe wavelengths used (530-nm pump and 490-nm probe).

**Results**: Without adaptive noise cancellation, the lock-in output primarily passes the laser RIN within its detection bandwidth, resulting in images that closely follow the linear transmissivity and lack sensitivity to pump-probe time delay. With adaptive noise cancellation in front of the lock-in, the RIN rejection is enough to restore the z-sectioning and sensitivity to pump-probe delay, as expected for transient absorption. Results were limited primarily by noise from the photodetector and analog-to-digital converter.

**Conclusions**: Digital adaptive noise cancellation, even when limited by electronics noise, can recover pump-probe signals by removal of laser RIN, under conditions where averaging alone fails.

## 1 Introduction

Pump-probe microscopy is an imaging modality that utilizes a femtosecond or picosecond pump laser pulse to prepare nonequilibrium electronic and vibrational states. Subtle changes of absorption of a probe pulse, arriving at a controlled delay after the pump, can track the evolution of transient states,^1^ thus providing for molecule-specific imaging contrast.^2^ Because this modality requires detection of extremely weak probe absorption changes, solid-state ultrafast laser sources, due to their low noise characteristic, are commonly employed.

Yet, fiber-based ultrashort laser sources could have significant advantages over bulk oscillators for transient absorption microscopy and techniques based on a similar detection principle, such as stimulated Raman scattering (SRS) microscopy,^3^ because of their permanent optical alignment, low cost, and compact size. However, the large laser relative intensity noise (RIN) of fiber-based laser sources increases the noise floor 20 to 30 dB above the shot-noise limit.^4^ In SRS and transient absorption microscopy, RIN is usually avoided by modulating the pump at high frequency and employing lock-in detection. This works well for bulk laser sources (e.g., Ti:sapphire) because the 1/f nature of the RIN makes it fall below the shot noise floor at MHz frequencies. But in fiber lasers, significant RIN that is 27∼40  dB higher than shot noise limit is found even at high-frequency components,^5^^–^^7^ making it difficult to reject with a lock-in. Compensating for such noise by slow scanning and long averaging at each pixel introduces other problems. This averaging strategy precludes high-speed imaging and is ineffective on 1/f noise.^8^ In addition, the heat deposited by long pixel dwell times can damage the sample. One widely used method to suppress laser RIN is balanced detection. By subtracting a matched reference from the signal, laser RIN is canceled, and the signal is preserved. However, it is difficult to keep the signal and reference balanced in laser scanning microscopy because the signal amplitude is rapidly modulated by the spatially varying transmissivity of the sample. To compensate these intensity fluctuations, several optical and electronic autobalanced detection methods have been developed.

a. In-line balanced detection (IBD)^9^ uses a birefringent plate to generate a time-delayed, orthogonally polarized, collinear replica of the signal. This replica, working as a reference, scans through the same sample area along with the signal. Thus, the intensity variation induced by the sample structure is the same for both the signal and the reference, automatically maintaining balance. After the sample, the probe and reference are separated by a polarizer, detected by separate photodetectors, and their photocurrents are subtracted electronically.
b. Collinear balanced detection (CBD),^10^ such as IBD, uses a time-delayed replica of the probe as the reference but needs only a single detector after the sample. In CBD, the delay is on the order of nanoseconds, so that in the radiofrequency Fourier domain, the probe and reference destructively interfere at the pump modulation frequency, automatically canceling the RIN.
c. Analog auto-balanced detection (AABD)^4^ samples a copy of the probe before the microscope as a reference. The probe and reference signals pass through an analog signal processing chain that uses a PID controller and a variable gain amplifier to balance the signals and cancel the RIN.

However, each of these methods has its tradeoffs. IBD and CBD expose the sample to twice the probe laser power, which increases the risk of photodamage. Moreover, both IBD and CBD rely on polarization to separate the reference and probe after sample, meaning they are not applicable to thick scattering or birefringent tissues. Furthermore, CBD only cancels RIN at a narrow radiofrequency band set by the probe-reference delay, limiting its potential for high-speed imaging, where rapid variations in pump-probe signal are spread across a radiofrequency band centered at the modulation carrier. AABD, on the other hand, does not expose the sample to the reference beam, does not rely on polarization, and cancels RIN across a bandwidth large enough for high-speed imaging. But AABD requires carefully designed, custom analog circuits, making it difficult to adapt and share among multiple laboratories.

In this work, we developed a software-based alternative to AABD, using digital adaptive noise cancellation (ANC). This method shares the advantages of AABD outlined above, including broad bandwidth RIN cancellation without the need to maintain orthogonally polarized probe and reference through the sample. Furthermore, digital ANC requires neither custom analog circuits as AABD does nor a dedicated hardware lock-in amplifier (LIA). The only signal processing hardware involved is a two-channel high-speed digitizer and a computer. The essentials of the signal processing chain, including ANC and LIA, can be conveyed in <20 lines of MATLAB code. In addition, digital ANC’s use of an adaptive finite impulse response (FIR) filter to shape the reference is more flexible than AABD’s use of a simple variable gain stage, in that the FIR filter can in principle compensate for differences in probe and reference photodetectors and suppress uncorrelated noise on the reference that does not contribute to noise cancellation. Our approach builds on previous works that demonstrated the ability to extract weak pump-probe signals from digital signals acquired by an analog-to-digital converter.^11^^,^^12^ The novelty here is in adding a digital adaptive filter before the lock-in.

In this paper, we demonstrate proof-of-concept pump-probe imaging of a test target exhibiting instantaneous nondegenerate two-photon absorption. We compare power spectral density (PSD) of the probe and the adaptive filter output to indicate the laser RIN reduction by the adaptive filter and will discuss limitations of the technique. Though in this experiment, the noise floor is set by the analog-to-digital converter, we conclude with a strategy for eventually reaching the shot noise limit.

## 2 Experimental Setup

**Fig. 1:**
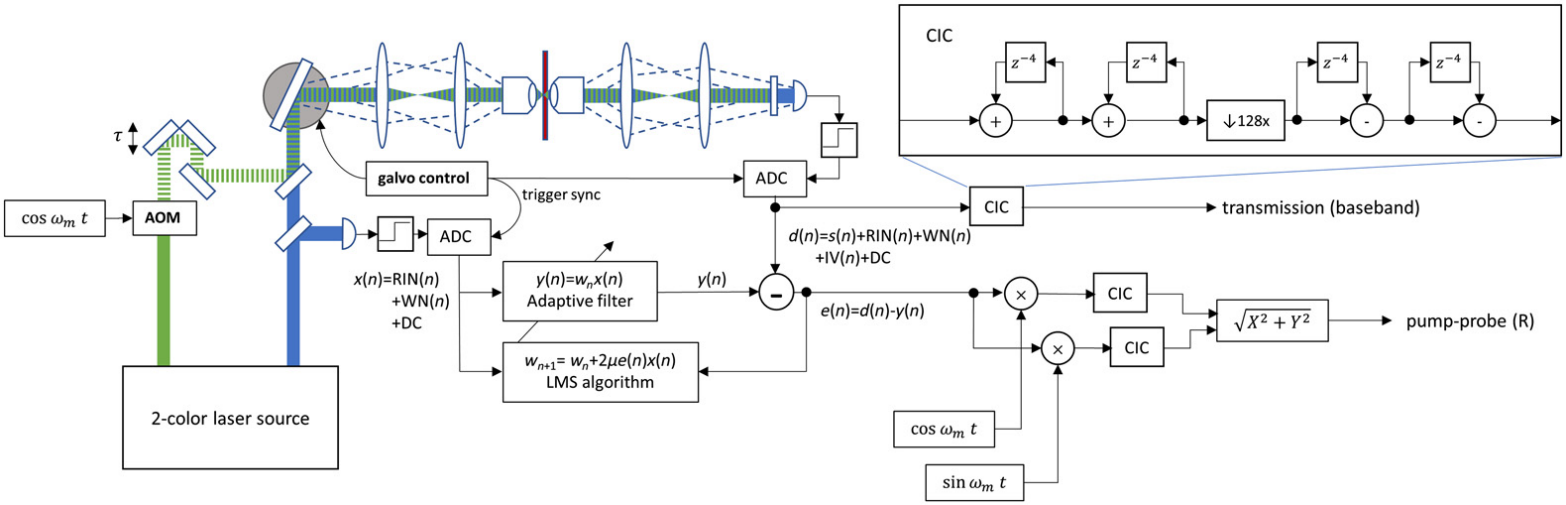
A conventional pump-probe microscope is employed to generate probe signal, d(n), and reference, x(n). The probe and reference signals feed into a software-based adaptive filter to produce the output e(n), which contains the pump-probe signal minus estimated RIN. Then, a software LIA and CIC filters are used to demodulate the pump-probe signal from the e(n). The transmission image is generated by directly applying the CIC filter to the probe d(n).

A schematic of the experimental setup is shown in Fig. 1. The fiber-based laser source used in this work is described in Ref. 13. In those prior experiments, a slow, stage-scanning microscope, and laser RIN were the major limitations of the instrument. We substitute the previous stage-scanner with a 3.5-kHz resonant scanner (EOPC). Successive lines are acquired by translating the sample with a stepper motor stage (ASI). In these experiments, the resonant scan amplitude is set so that the beam scans across a 75-μm line on the specimen. We use an 8-mW pump at 530 nm and an 8-mW probe at 490 nm, pump modulation frequency of $f_{m}=1\;\mathrm{MHz}$, and laser repetition rate of 65 MHz. The pump and probe pulse durations are ∼1  ps, as estimated by cross-correlation [Fig. 5(a)].

Before entering the microscope, the probe beam is split by a 50/50 beam splitter. One half of the probe is detected directly by a photodetector (PDA36A, Thorlabs) to serve as the reference signal. The other half is transmitted through the microscope and then detected by an identical photodetector to serve as the probe signal. These pass through 22-MHz low-pass antialiasing filters (Minicircuits) and are digitized by the two 50-MHz ADC channels of a data acquisition (DAQ) device (Analog Discovery Studio, Digilent). The ADC is synchronized to the resonant scanner through a TTL trigger.

All digital signal processing is done in MATLAB. The transmitted light image is formed by passing the probe d(n) through a downsampling cascaded integrator comb (CIC) filter,^14^ which reduces the sample rate from 50 MHz to the pixel rate of 2.56  μs/pixel. The pump-probe image is formed by passing the probe d(n) and reference x(n) through an adaptive filter and then a LIA. Lock-in X and Y channels pass through identical CIC filters to low-pass and downsample to the pixel clock, and the magnitude R is calculated as the final output (limitations with the DAQ digital input buffers prevented simultaneous acquisition of a synchronization signal for $f_{m}$). The MATLAB code for processing each line is sketched below (some details omitted for brevity).

% setup

decimator = dsp.CICDecimator(128,4,2);

fmod = 1.0e6; % modulation frequency 1 MHz

nco_i = cos(2*pi*fmod*t).'; % in-phase NCO

nco_q = sin(2*pi*fmod*t).'; % quadrature NCO

adaptFilt = dsp.LMSFilter(8,'Method','LMS','StepSize',0.1);

…

% processing for each line scan

d = % fetch data from ADC probe channel

x = % fetch data from ADC ref channel

decimator.reset();

trans = decimator(d); % transmitted light image

[y,e] = adaptFilt(x,d); % apply adaptive filter

% lock-in

prod_i = e.*nco_i;

prod_q = e.*nco_q;

decimator.reset();

lia_x = decimator(prod_i);

decimator.reset();

lia_y = decimator(prod_q);

lia_r = sqrt(lia_x.^2 + lia_y.^2);

## 3 Theory and Background

### 3.1 Signal and Noise Sources in Transient Absorption Microscopy

To interpret our results and understand the limits of adaptive noise canceling, we first outline the various signal and noise sources. This discussion is inspired by the detailed account of signal and noise in coherent Raman spectroscopy by Zhang et al.;^15^ our focus, rather than comparing SNR of different techniques, is to highlight signal and noise sources in terms of their handling by the signal processing chain. An additional resource for signal and noise considerations in transient absorption microscopy can be found in the review by Fischer et al.^2^ We start by considering the optical signal and noise sources, adding electronic noise (amplifier, ADC) later. The photocurrent generated from the transmitted probe intensity through the sample, $i_{0}(t)$, can be expressed as

$$i_{0}(t)=Rp_{0}(t)[1+\sigma (t,\tau )p_{\mathrm{pu}}(t)]t_{s}(t)+i_{\text{shot}}(t)\;=Rp_{0}(t)t_{s}(t)+Rp_{0}(t)p_{\mathrm{pu}}(t)[\sigma (t,\tau )t_{s}(t)]+i_{\text{shot}}(t),$$ (1)

where R is the photodiode responsivity, $p_{0}(t)$ is the incident probe beam power, σ(t,τ) is the pump-probe interaction cross-section, $p_{\mathrm{pu}}(t)$ is the modulated pump power, $t_{s}(t)$ is the sample transmissivity, and $i_{\text{shot}}(t)$ is the contribution from shot noise. To first order, two of these vary with respect to laser scan position and ability to carry information about the sample: σ(t,τ) and $t_{s}(t)$. These are related to sample spatial coordinate $\vec{r}$ through a function of the scan pattern, $g:t\to \vec{r}$. The $p_{0}(t)$ probe power contains both a DC component $p_{\mathrm{DC}}$ and laser RIN $p_{\mathrm{RIN}}(t)$. By comparison with the expressions for SRS in Ref. 15, Eq. (1) has a number of important differences. We neglect, for the moment, electronics noise sources [to be included in Eq. (5)] and refer to laser noise as “RIN” to allow for non-1/f noise spectra. We explicitly consider the dependence of the pump-probe cross-section $\sigma \propto \chi^{\left(3\right)}$ on spatial coordinate $\vec{r}$ and pump-probe time delay τ. We also have added spatial dependence of sample transmissivity $t_{s}$, which, under high-speed scanning, can produce appreciable overlap with the lock-in detection band, which manifests as a τ-independent background signal [see Figs. 6(j), 7, and related discussion]. The effect of this rapidly varying transmissivity, in the case of nonimaging pump-probe spectroscopy, has earned the label “catastrophic noise,” which can be mitigated by discarding measurements through histogram filtering.^16^ However, in the case of imaging microscopy, such a filtering method would discard pump-probe signal around edges and highly textured regions of the sample.

In the frequency domain, we can rewrite Eq. (1) as

$$I_{0}(f)=RP_{0}(f)⊗T_{s}(f)+RP_{0}(f)⊗P_{\mathrm{pu}}(f)⊗\Sigma (f,\tau )⊗T_{s}(f)+I_{\text{shot}}(f),$$ (2)

where $P_{0}$, $T_{s}$, Σ, $P_{\mathrm{pu}}$, and $I_{\text{shot}}$ are the Fourier transforms of the probe power, sample transmissivity, pump-probe cross-section, pump power, and shot noise, respectively, and ⊗ represents convolution. After substituting $P_{0}(f)=P_{\mathrm{dc}}\delta (f)+P_{\mathrm{RIN}}(f)$ and $P_{\mathrm{pu}}(f)=P_{0,\mathrm{pu}}\;\delta (f-f_{m})$, where $f_{m}$ is the pump modulation frequency, we can rewrite the photocurrent generated from probe intensity in Fourier domain as follows:

$$I_{0}(f)=RP_{\mathrm{dc}}T_{s}(f)+RP_{\mathrm{RIN}}(f)⊗T_{s}(f)+RP_{\mathrm{dc}}P_{0,\mathrm{pu}}\delta (f-f_{m},\tau )⊗[\Sigma (f,\tau )⊗T_{s}(f)]+I_{\text{shot}}(f).$$ (3)

We have neglected the product between probe RIN and the pump, as it is significantly weaker than the other terms. Only the third term contains the desired pump-probe signal, Σ(f,τ), which is to be demodulated by the LIA. The first term $\propto T_{s}$ conveys the linear transmissivity, and its bandwidth depends on the line scan rate, diffraction-limited spot size, scan area, and the spatial frequency extents of the sample. This term can be rejected with large $f_{m}$ and lock-in detection, but for high-speed scanning, $T_{s}(f)$ can overlap with the lock-in band, resulting in residual background signals in the pump-probe measurement [see Figs. 6(j), 7, and related discussion]. The last term, $I_{\text{shot}}$, can be mitigated by having sufficient pump-probe power product for an acceptable signal-to-shot noise ratio:

$${\mathrm{SNR}}_{\text{shot}}\propto \frac{P_{\mathrm{dc}}P_{0,\mathrm{pu}}}{I_{\text{shot}}}\propto \frac{P_{\mathrm{dc}}P_{0,\mathrm{pu}}}{\sqrt{P_{\mathrm{dc}}}}\propto \sqrt{P_{\mathrm{dc}}}\;P_{0,\mathrm{pu}}.$$ (4)

Thus, SNR is improved either by increasing pump power, probe power, or both. But increasing pump power will have a stronger effect on SNR because of the deleterious effects of probe shot noise.

The second term in Eq. (3), $R\;P_{\mathrm{RIN}}(f)⊗T_{s}(f)$, usually handled by modulating the pump at a frequency where RIN is absent.^17^^,^^18^ However, for fiber-based laser sources with non-1/f, broad-spectrum noise,^13^ it is impossible to find a lock-in detection band absent of RIN. When transmitted probe RIN leaks into the lock-in detection band, the net effect is to contaminate the transient absorption image with the linear transmitted light image (see Fig. 6). This effect worsens with scanning speed, as the increased lock-in detection bandwidth gathers more RIN. Effective RIN cancellation can be done with balanced detection^4^^,^^9^^,^^10^ (referred to as ratiometric detection in low repetition rate experiments^11^). To cancel this term with balanced detection, the reference must be modulated to match the time-varying sample transmissivity $T_{s}$, imposing a serious limitation on imaging speed without custom electronics.^4^^,^^19^ Gambetta et al. utilized an unmodulated reference to achieve the conventional balanced detection for SRS signal enhancement. However, because of the persistence of the intensity mismatch between reference and signal, a 500-ms integration time is required to attain the desired sensitivity.^19^ This RIN term is what we seek to eliminate with digital ANC.

Next, we consider electronic noise sources. The built-in photodiode transimpedance amplifier converts the overall photocurrent $I_{0}(f)$ to voltage. The transimpedance amplifier and the analog-to-digital conversion contribute additional noise. Thus, in the frequency domain, the overall signal before the adaptive filter can be expressed as follows (assuming a 50-Ω load):

$$V_{0}(f)=I_{0(f)}\times 50\;\Omega +V_{\mathrm{amp}}(f)+V_{\mathrm{ADC}}(f)\;=\{RP_{\mathrm{dc}}T_{s}(f)+RP_{\mathrm{RIN}}(f)⊗T_{s}(f)+RP_{\mathrm{dc}}P_{0,\mathrm{pu}}\delta (f-f_{m},\tau )⊗[\Sigma (f,\tau )⊗T_{s}(f)]+I_{\text{shot}}(f)\}\times 50\;\Omega +V_{\mathrm{amp}}(f)+V_{\mathrm{ADC}}(f).$$ (5)

By comparison with the expressions for SRS in Ref. 15, Eq. (5) has an additional noise term to account for the ADC and does not yet consider LIA gain. These broad-spectrum, uncorrelated electronic noise sources can be minimized in low repetition-rate experiments using gated acquisition to reject noise in between probe pulses.^11^ For high repetition-rate experiments, this term can be minimized using a long time constant on the lock-in to restrict bandwidth,^15^ but this comes at the expense of limiting imaging speed.

**Fig. 2:**
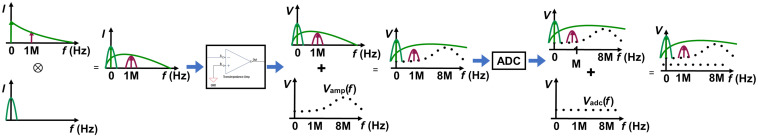
Signal and noise processes in transient absorption microscopy: (a) DC, 1 MHz, and RIN convolve with sample structural baseband to produce the 1-MHz pump-probe signal contaminated by laser RIN. (b) The superimposed electronic noise from transimpedance amp of the photodetector further degrades SNR. (c) Adding ADC electronic noise floor directly to generate the overall noise floor. The overall noise floor prohibits the shot noise-limited detection.

A summary of these processes is sketched in Fig. 2. After superposition of all the noise sources, the pump-probe signal, marked as red, becomes buried underneath the unwanted components.

A balanced detector can eliminate noise that is correlated between the signal and reference arms (common-mode noise) such as laser RIN. When the electronic noise floor is below the shot noise floor, a balanced detector will reduce the overall noise floor to 3 dB above the shot noise floor.^20^ This limit arises from the separate shot noise contributions of the two detectors. Unlike the RIN, these add together at the balanced detector’s output because they are uncorrelated. Likewise, electronic noise (photodiode amplifier noise, ADC input noise) is also uncorrelated. Therefore, when the electronic noise floor is above the shot noise floor, a balanced detector will reduce the overall noise floor to 3 dB above the electronic noise floor. We take this theoretical limit into consideration when tuning the adaptive filter’s performance.

### 3.2 Digital Adaptive Filtering

A digital adaptive filter, in the configuration shown in Fig. 1, searches for filter coefficients that transform the reference, x→y, to match the desired probe signal, d.^21^ In this work, we use an LMS adaptive filter, which is an FIR filter that adjusts its coefficients to minimize mean-square error between d and y through gradient descent (we refer readers to any textbook on adaptive signal processing, e.g., Ref. 22, for details). In a noise canceling configuration, d is a noisy signal and x is an independent sampling of the noise. The filter output y estimates the noise component of d and subtracts the two to produce an estimate of the underlying signal, e. This technique has been widely used in areas such as telephone echo cancellation, noise cancellation, equalization of communication channels, active noise control, and adaptive control systems.^22^ In our experiment, the laser RIN common to the probe (d) and reference (x). is correlated, allowing the adaptive filter to produce an estimate of the laser RIN in the probe (d) for direct cancellation.

**Fig. 3:**
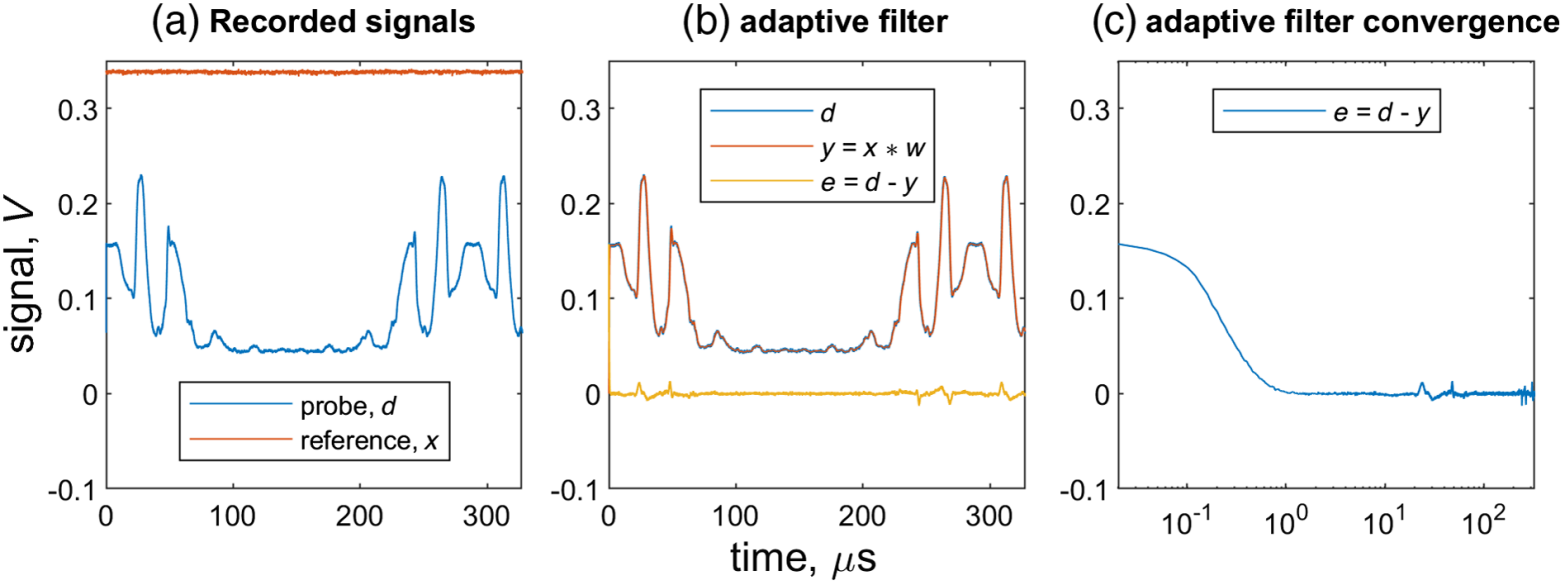
(a) Acquired reference and probe signals from one line scan across the BGO particles. (b) The adaptive filer alters x to become y, tracking the amplitude of d. Subtraction of y from d produces the adaptive filter output, e. (c) Plot of e with respect to logarithmic time scale.

Figure 3 shows typical inputs and outputs the digital adaptive filter. Figure 3(a) shows the measured reference x(n) and probe d(n) a line scan across BGO crystal particles, covering both forward and backward motion of the resonant scanner. Figure 3(b) shows that the adaptive filter’s transformation of the reference x(n) to y(n) tracks the probe amplitude d(n). The difference, e(n), contains the pump-probe signal, with RIN canceled, plus some residual tracking error. Figure 3(c) plots e(n) with time on a logarithmic scale, showing convergence in about 1  μs.

**Fig. 4:**
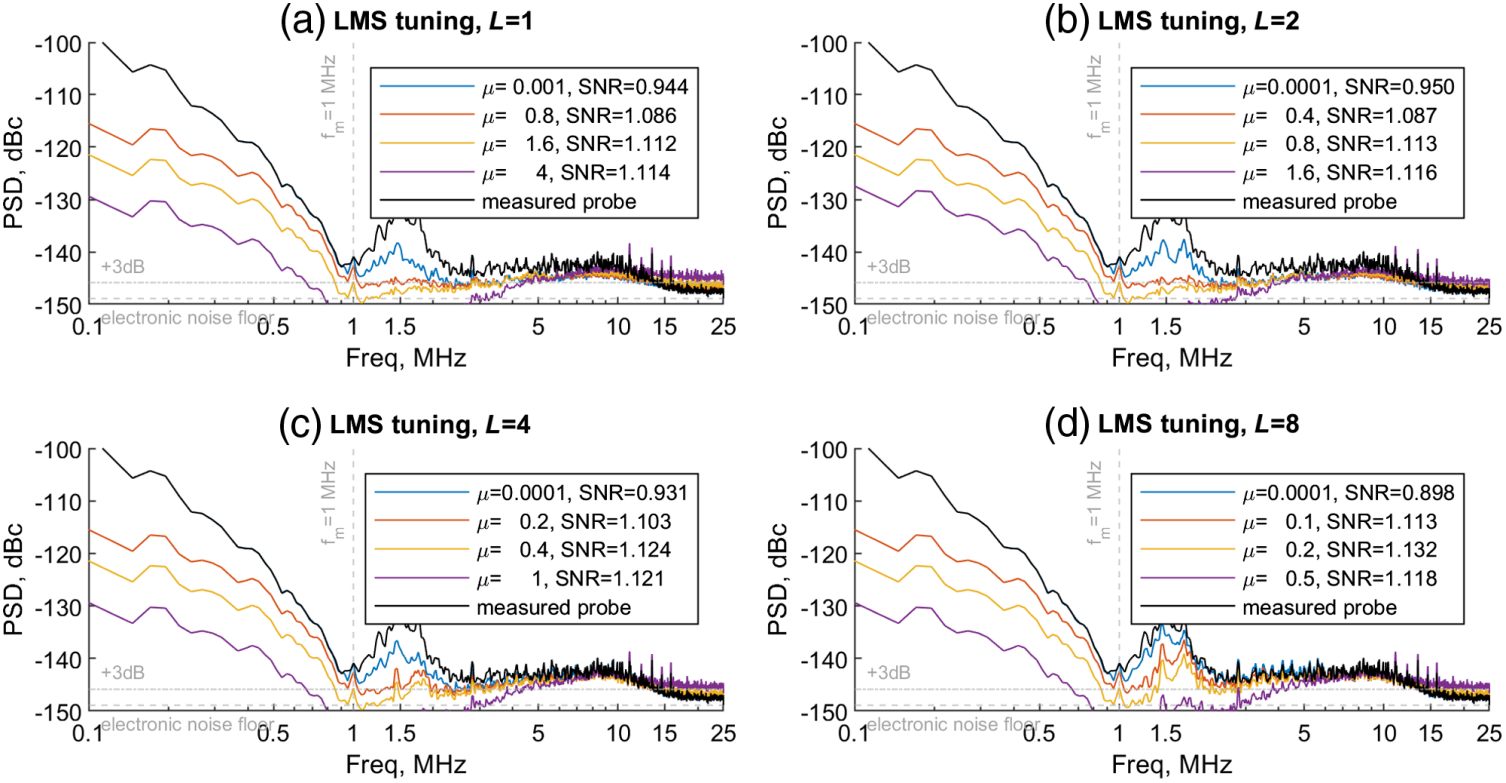
ANC-enhanced PSDs for BGO particle imaging experiments. PSDs shown are max projections across all scan lines of the image, for a single repetition. The adaptive filter noise canceling performance is evaluated with respect to different filter lengths L and step sizes μ.

For a given adaptive filter length L, we select a step size μ such that the noise floor of e=y−d is at 3 dB above the electronics noise floor (the theoretical limit for an electronic noise-limited balanced detector, as discussed in Sec. 3.1), which we estimate from the power spectrum at f>15  MHz, above the photodetector cutoff. This turns out to be μ≈0.8/L for our conditions. As can be seen in Fig. 4, larger μ has an advantage in tracking transmitted probe intensity more closely, but also erodes the signal and increases high-frequency noise. This increased noise for large μ is also a symptom of the LMS gradient descent overshooting and oscillating about the optimum filter coefficients.^22^ The best SNR enhancement appears to be at L=8 and μ=0.1. Filters with L≥16 (not shown) were found to be less stable under our conditions and fail to further enhance SNR.

### 3.3 Sampler Bit Width, Oversampling, and Voltage Resolution

An important consideration is whether the ADC provides enough quantization steps to resolve weak pump-probe signals.^12^ We use a 14-bit ADC, operating in a ±1  V range, resulting in a quantization step of ∼122  μV. A CIC filter with R=128, M=4, N=2 is used for decimation. The total bit width of the decimated result is given as^14^

$$B_{\max}=N\;\log_{2}\;RM+B_{\text{in}}-1,$$ (6)

where $B_{\text{in}}$ is 14-bit input from the ADC. This oversampling approach allows us to expand the dynamic range to 31 bits for pump-probe measurement, resulting in effective ∼0.93  nV resolution.

## 4 Results

**Fig. 5:**
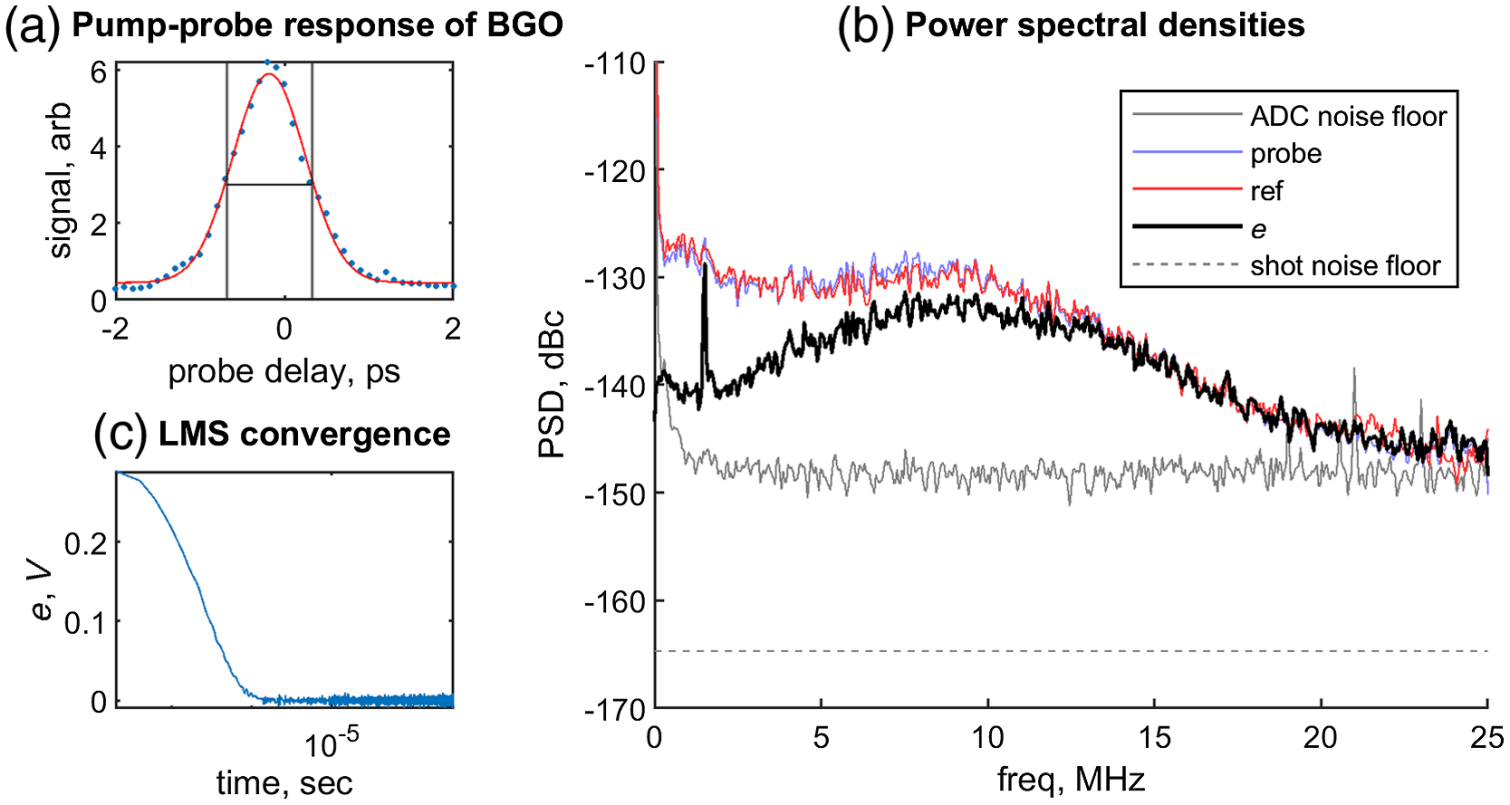
(a) (blue dots) Measured pump-probe response of BGO at 530-nm pump, 490-nm probe, (red line) fit to Gaussian with 1 ps FWHM. (b) The measured PSDs of probe d, reference x, adaptive filter output e, and ADC noise floor are shown. Electronic noise floor is 24 dB above shot noise limit and ANC shows noise reduction around 1 MHz region. (c) The adaptive filter converges after 1  μs.

We show results progressing from (1) line scans through a uniform sample to (2) imaging a spatially heterogeneous sample. For a specimen, we use ${\mathrm{Bi}}_{4}{\mathrm{Ge}}_{3}O_{12}$ (BGO, available from MTI Corporation, Richmond, California), a crystal with a bandgap of 4.36 eV (284 nm).^23^ For our 530-nm pump (2.34 eV) and 480-nm probe (2.58 eV), both of which are below the bandgap individually, but above the bandgap at the sum of their frequencies, we expect to see only a nondegenerate two-photon absorption. Consistent with this, the pump-probe delay scan [Fig. 5(a)], acquired with a commercial LIA (SR844, Stanford), indicates an instantaneous increase in probe absorption centered at time overlap (τ=0). This scan also reveals our pump-probe cross-correlation duration to be 1 ps.

### 4.1 Single Scan Line, Uniform Sample, and Power Spectral Density Results

Figure 5(b) shows PSD plots of the transmitted probe (d), reference (x), the noise canceling output of the adaptive filter (e), along with the ADC noise floor and calculated shot noise limit. (These data were acquired on a single, uniform, BGO crystal, and so the LMS parameters L=3, μ=0.2 were set differently than for the case of a heterogeneous structured sample.) PSD units are in decibels relative to the carrier (dBc), referenced to the DC power of the reference ($\propto 0.1225\;V^{2}$). As can be seen from probe and reference PSDs, the ADC noise floor is about 15 dB above the shot noise limit and the laser RIN contributes an additional ∼20  dB to the overall noise floor. A 1/f character of the RIN can be seen from DC to 1 MHz. Above 1 MHz, a gradual increase starts to appear, hitting a maximum at 8 MHz, which we attribute to the photodetector transimpedance amplifier.^24^ Above 15 MHz, the noise from both probe and reference photodetectors converges to the ADC noise floor.

**Fig. 6:**
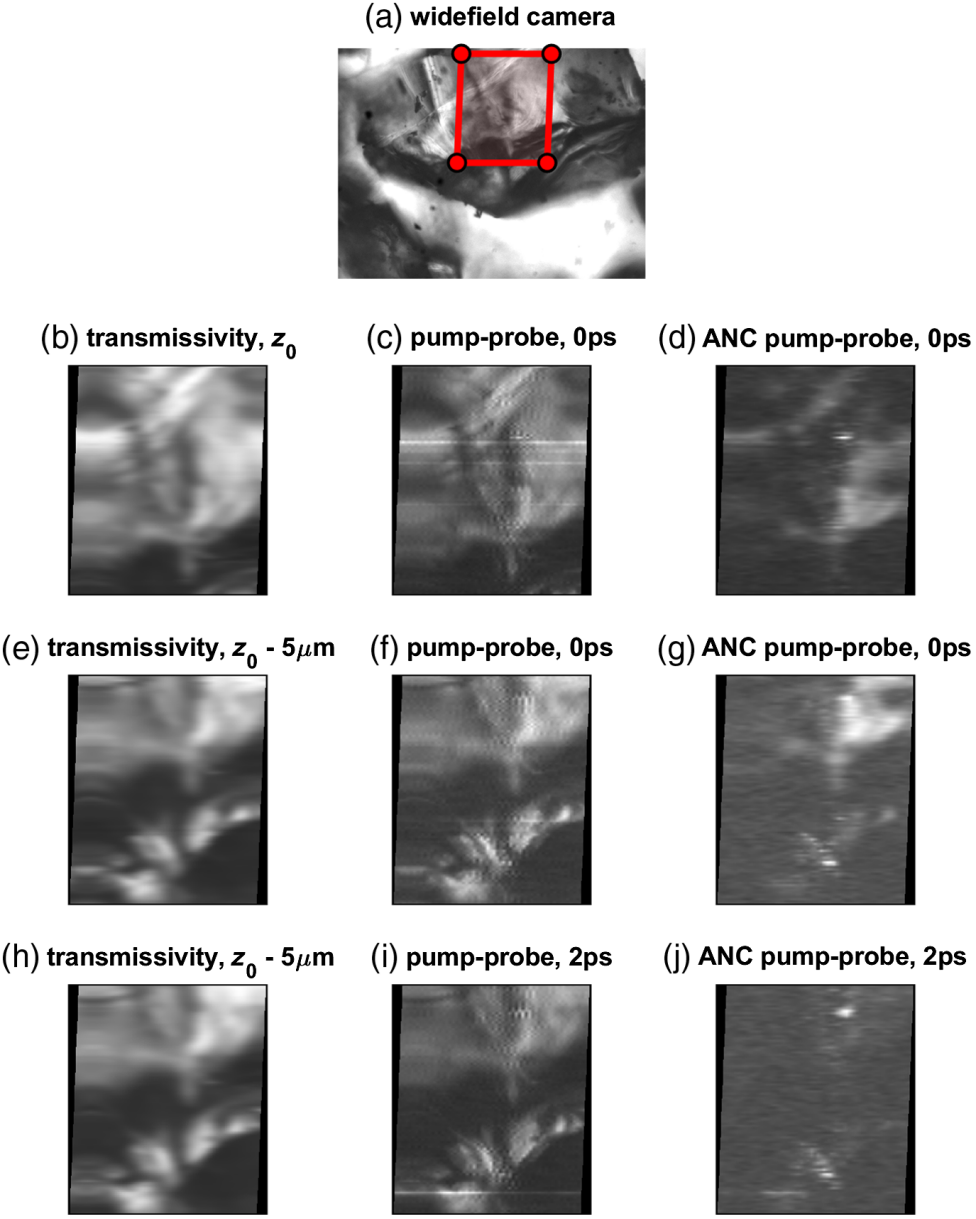
Imaging results. (a) The wide-field transmission image is shown on the top, with laser scanned area (70  μm×100  μm) highlighted by the red box. (b) Laser-scan transmissivity, (c) lock-in output without ANC, and (d) lock-in output with ANC, at 0-ps pump-probe delay. (d) Laser-scan transmissivity, (e) lock-in output without ANC, and (f) lock-in output with ANC, for a z-section offset by 5  μm. (h) Laser-scan transmissivity, (i) lock-in output without ANC, and (j) lock-in output with ANC, for 2-ps pump-probe delay.

**Fig. 7:**
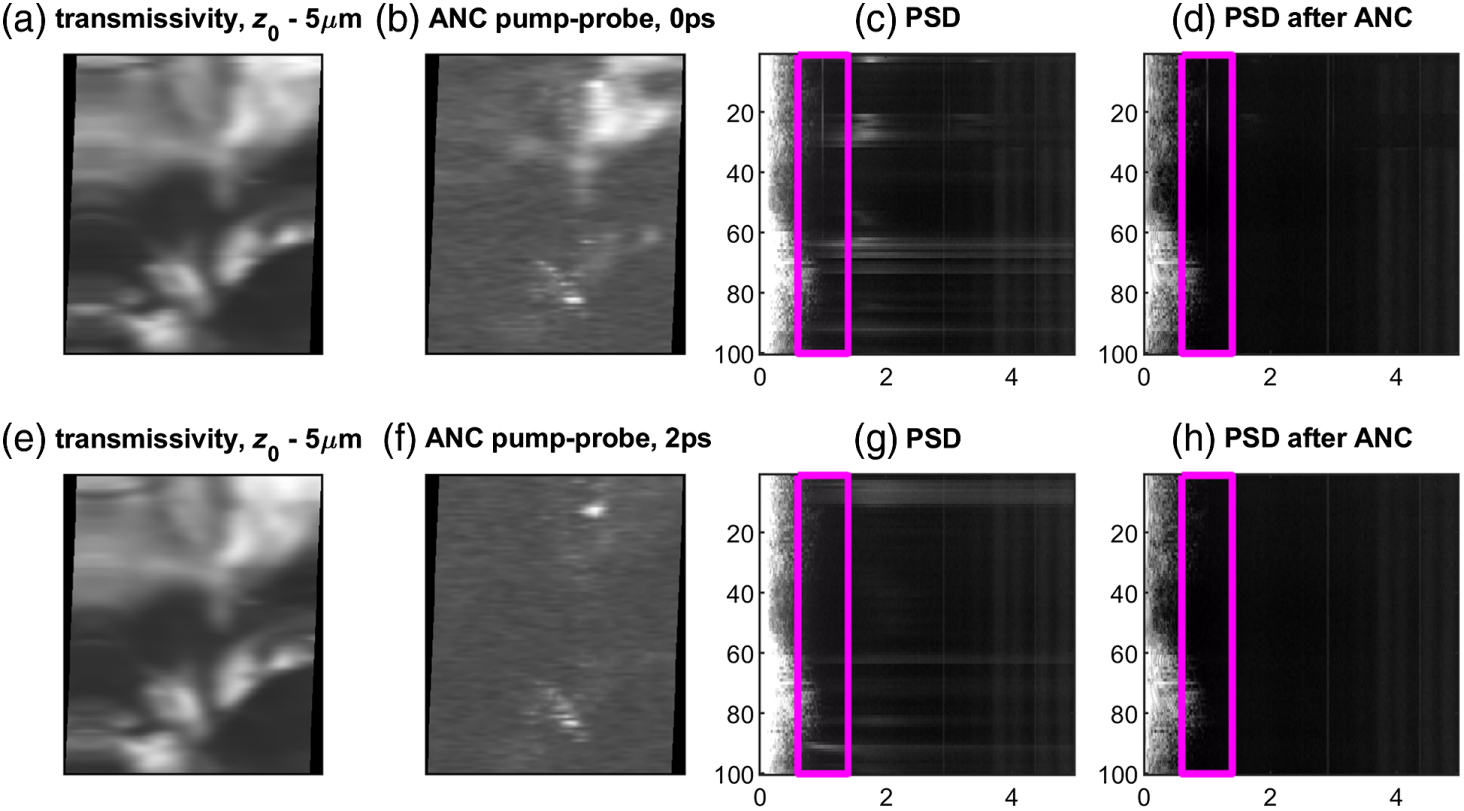
Imaging results along with power spectral densities of each scan line. (a) Laser-scan transmissivity, (b) lock-in output with ANC, (c) PSD without ANC, and d) PSD with ANC for 0-ps pump-probe delay. Note the pump-probe signal at 1 MHz in both PSDs. (e) Laser-scan transmissivity, (f) lock-in output with ANC, (g) PSD without ANC, and (h) PSD with ANC for 2-ps pump-probe delay. Note the absence of pump-probe signal at 1 MHz in both PSDs.

When inspecting the PSD of adaptive filter output e, the noise floor in the 1-MHz region has been decreased and the 1-MHz pump-probe signal can be seen clearly. The total noise reduction in the vicinity of this signal is ∼10  dB. This highlights that the electronic noise is currently the limiting factor, and that additional averaging is required to see a clear pump-probe image. We show later that averaging alone is not sufficient to recover a pump-probe image under these conditions (Figs. 6 and 7), but first we discuss what it will take to reach the shot noise limit and estimate how much averaging is needed in the meantime.

From Fig. 5, we notice that the superimposed noise floor of the ADC and the photodetector (shown as the black solid curve) is 24 dB larger than the shot noise limit. This shot noise limit is equivalent to $27\;\mathrm{pW}/\sqrt{\mathrm{Hz}}$. By comparison, the NEP of our photodetector is $100\;\mathrm{pW}/\sqrt{\mathrm{Hz}}$ and the ADC raises the NEP of the entire detection system to $1000\;\mathrm{pW}/\sqrt{\mathrm{Hz}}$. We note that commercial systems that incorporate a low-noise transimpedance amplifier and ADC can reach NEP of $10\;\mathrm{pW}/\sqrt{\mathrm{Hz}}$ (e.g., DPD80, Resolved Instruments), indicating that digital adaptive RIN canceling can in principle achieve shot noise-limited detection with a suitable low-noise detector and ADC.

### 4.2 Imaging Results

Here, we perform pump-probe imaging, with and without ANC, on BGO crystal particles. The specimen was prepared by crushing a fragment of BGO on a glass slide with a metal pick. Then, a spacer was placed around the particles, with a coverslip on top. Images were acquired with 100× line repetitions. Subsequent lines were acquired by stepping the sample stage in 1-μm increments. Given the 50-MHz sample rate (20  ns/sample) and 128× downsampling after the lock-in mixers, this 100× averaging is equivalent to a lock-in integration time constant of 256  μs. If, instead of translating the sample on the y axis, we were to use the 2-Hz frame rate y-scanner on the EOPC system and implement the signal processing chain on a real time DSP processor or FPGA (future work), including forward and backward scans, this 100× averaging time would translate to a 25-s/frame pump-probe frame rate. The field of view is 75  μm×100  μm.

Two images were acquired at 0 ps delay, at z-sections 5  μm apart. The third image was acquired at the lower z-slice, with 2 ps delay. As the delay scan in Fig. 5(a) indicates, we expect a strong pump-probe signal at 0 ps, and no signal at 2 ps delay. We will show this is the case only after adaptive filtering. Results are shown in Fig. 6 for transmitted light (no averaging of line scans), 100× averaging of the conventional lock-in, and 100× averaging of lock-in after noise canceling.

Without adaptive filtering (Fig. 6, center column), the pump-probe output merely follows the transmitted intensity, with only a slight reduction in brightness at 2 ps delay. In this scenario, the lock-in primarily detects laser RIN within its bandwidth; the result is a noisy copy of the transmitted light image. On the other hand, with adaptive filtering (Fig. 6, right column), the RIN is reduced and the lock-in algorithm produces pump-probe images that are sensitive to z-section, and also sensitive to pump-probe time delay, as expected. We note in Fig. 6(j) that, even though BGO is not expected to have long-lived excited states for our pump/probe wavelengths, there is still a signal at 2 ps. We attribute this to a τ-independent background caused by leakage of the linear transmitted image $T_{s}$ into the lock-in detection band, as will be discussed next.

Figure 7 shows the z=−5  μm results along with corresponding PSD of the signal from each line, just before lock-in detection. The 1-MHz pump-probe signal is clearly present in the PSD plot of the 0 ps delay image and disappears at 2 ps delay, as expected for BGO [see Fig. 5(a)]. It can also be seen that the background within the lock-in bandwidth (magenta box) is much cleaner after ANC. Finally, we observe leakage of the linear transmission image $T_{s}$ (centered at 0 MHz on the spectrograms) into the lock-in band (magenta box) in Figs. 7(c), 7(d), 7(g), and 7(h). This is especially pronounced around scan lines 15 and 70 to 85, where the presence of signal within the lock-in detection band results in bright spots even in the 2 ps image [Fig. 7(f)], even though there is no pump-probe signal. This can potentially be resolved by increasing the pump modulation frequency $f_{m}$, slowing the scanner, or using RF phase-sensitive lock-in (the lock-in for these experiments was outputting the phase-insensitive magnitude; limitations with the DAQ digital buffers prevented simultaneous acquisition of a pump synchronization signal).

## 5 Discussion and Conclusions

In summary, we have demonstrated that performing ANC and lock-in detection on digitized photodetector streams from a pump-probe microscope can recover a transient absorption signal when averaging alone is insufficient.

Our current setup is limited in that it is slow (the imaging experiments here took 6 h each), lacks phase-sensitive synchronization between the pump modulation and the lock-in, and is far from achieving shot noise-limited detection. Because of these limitations, we opted to perform proof-of-concept imaging here on a sample with a strong two-photon absorption response, rather than biological targets such as heme proteins. However, the results do show that an LMS adaptive filter can reduce the laser RIN in the presence of spatial structure on par with cells and tissues. The first two problems will be addressed in future work by either using a DAQ with a large enough buffer and data transfer bandwidth to support real-time streaming of both the ADC and digital synchronization signals to the host computer, or by implementing the signal processing chain in real time on an FPGA.^12^ Even without reaching the shot noise limit, we estimated this approach will achieve frame rates of about 2  frames/s. The third problem, reaching the shot noise limit, can be addressed in future work by an appropriate selection of photodetector, transimpedance amplifier, and ADC that brings the electronics noise below the shot noise floor. Suitable commercial detectors exist, with integrated photodiode, amplifier, and ADC.

In conclusion, we expect that digital ANC will enable shot noise-limited transient absorption microscopy with high frame rates from inexpensive fiber laser sources. We anticipate this technique will also find use in other microscopies that rely on transferring modulation from one beam to another, such as SRS and photothermal microscopy. This software-based technique is far easier to implement and customize than approaches that rely on analog RF signal processing circuits. In addition, this work sets the stage for more sophisticated adaptive filters and potentially neural networks for pump-probe signal preprocessing.

## Code, Data, and Materials Availability

Code and data associated with this manuscript can be requested by contacting the corresponding author (JWW). Availability of materials has been marked within the manuscript.
